# Motivational Interviewing to Improve the Uptake of Colorectal Cancer Screening: A Systematic Review and Meta-Analysis

**DOI:** 10.3389/fmed.2022.889124

**Published:** 2022-04-26

**Authors:** Novia Niannian Long, Michele Petrova Xin Ling Lau, Ainsley Ryan Yan Bin Lee, Natalie Elizabeth Yam, Nicholas Ye Kai Koh, Cyrus Su Hui Ho

**Affiliations:** ^1^Yong Loo Lin School of Medicine, National University of Singapore, Singapore, Singapore; ^2^Department of Psychological Medicine, Yong Loo Lin School of Medicine, National University of Singapore, Singapore, Singapore

**Keywords:** cancer screening, preventative medicine, behavioral science, colorectal cancer, motivational interviewing, psychology, systematic review

## Abstract

**Introduction:**

Colorectal cancer screening when done early can significantly reduce mortality. However, screening compliance is still lower than expected even in countries with established screening programs. Motivational interviewing is an approach that has been explored to promote behavioral change including screening compliance. This review synthesizes the efficacy of motivational interviewing in promoting uptake of colorectal screening modalities and is the only review so far that examines motivational interviewing for colorectal cancer screening alone.

**Methods:**

A systematic review and meta-analysis was conducted to examine the effects of motivational interviewing for colorectal cancer screening. PubMed, EMBASE, CENTRAL, PsycINFO, and CINAHL were searched to identify eligible studies from inception to June 2021 and selection criteria was defined. Risk of bias was assessed using the Cochrane Risk of Bias 2.0 tool. The DerSimonian and Laird random effects model was used in the statistical analysis for studies included in the meta-analysis.

**Results:**

Fourteen studies from 14 randomized-controlled trials with a low to moderate risk of bias were analyzed. 8 studies in the systematic review showed that motivational interviewing is superior to a control group. Meta-analysis was conducted on 11 studies and showed that motivational interviewing is statistically significant in increasing colorectal cancer screening rates in both intention-to-treat and per-protocol analysis. Timing of data collection of colorectal cancer screening rates did not make a significant difference to the efficacy of motivational interviewing. Studies that offered and accepted a mixture of colorectal screening modalities such as colonoscopy and fecal immunochemical tests were significantly more likely to have favorable colorectal screening outcomes. Heterogeneity in intervention was noted between studies, specifically differences in the training of interventionists, intervention delivery and comparator components.

**Conclusion:**

Motivational interviewing is a tailored intervention demonstrating mixed evidence in improving colorectal cancer screening attendance amongst individuals. More research is needed to rigorously compare the effect of motivational interviewing alone vs. in combination with other screening promotion strategies to enhance colorectal cancer screening compliance.

## Introduction

Colorectal cancer (CRC) is the third most common cancer and second most common cause of cancer death worldwide (1). Appropriate screening can ensure early detection of CRC, allowing elimination of adenomas via polypectomy to prevent further progression of disease (2, 3). Moreover, screening programs for CRC are widely available worldwide, making it easily implementable.

Despite its benefits, screening rates continue to remain low even in countries with established screening programs. A survey conducted in Singapore in 2015–2016 showed that only 27.3% eligible respondents screened for CRC within the recommended period (4). Similarly, in a study conducted in the United States in 2015, 63.4% of women and 61.9% of men reported having a recent CRC screening, falling short of the government’s target of 80% of its eligible population by 2018 (5). Several barriers could be attributed to these low numbers. Common reasons for the aversion to such health check-ups include a lack of awareness, time constraints, difficulty in access to the services and negative experiences and emotions associated with CRC screening, among others (6).

Therefore, it is worth exploring interventions that can help boost patient participation in CRC screening to improve upon existing screening programs. Much literature has provided evidence to support the effectiveness of various health promotion strategies to advocate for CRC screening, such as mailed media prints, invitation letters and reminder calls (7–10). However, individuals approach change with varying degrees of readiness (11) and may sometimes require more rigorous, individualized efforts to build motivation and reduce resistance. One of such approaches is motivational interviewing (MI) which is a collaborative person-centered counseling approach initially developed to address alcohol addiction (12). Practitioners of MI establish rapport with patients to explore their motivation and ambivalence for screening, rolls with resistance and evokes patient’s own reasons to implement behavioral change (13, 14). Instead of confrontation or persuasion, MI minimizes resistance and builds self-efficacy for behavioral change in a more sustainable manner (11). In fact, MI has been researched and explored for its effectiveness in health behaviors (11, 14, 15) and has shown promise in promoting diet and exercise, diabetic control, oral health, weight loss and more optimal blood pressure (16, 17).

To date, there are no systematic reviews or meta-analysis looking at the effectiveness of MI on CRC screening uptake alone. Unlike screening for other cancers, eligible patients for CRC screening encounter more barriers (18). CRC screening involves many different screening modalities such as colonoscopy, barium enema, sigmoidoscopy, fecal occult blood test (FOBT), each with its own test-specific barriers such as bowel preparation and feelings of disgust (19). Therefore, this systematic review and meta-analysis aimed to evaluate the role of MI, either as a stand-alone intervention or integrated with other strategies, for the promotion of CRC screening specifically. It also aimed to provide recommendations for future researchers, policymakers, and healthcare providers regarding theory-based approaches to incorporate MI for CRC screening promotion.

## Materials and Methods

### Selection Criteria

All randomized-controlled trials (RCTs) were included if they fulfilled the following:

• Implemented an intervention involving MI as part of any arm, delivered in any format (e.g., face-to-face, telephone).

• Included colorectal screening participation as an outcome.

• Included participants who were eligible for CRC screening.

• Involved a comparator arm that did not involve MI.

• Article is written in English.

All RCTs included in the systematic review were also included in the meta-analysis if they:

• Involved a comparator arm that did not involve any components of MI nor tailored counseling.

• Recorded and reported participation rate in CRC screening as an outcome as opposed to changes in CRC screening beliefs, intents or adherence rate.

• Accepted any screening modalities as a screening outcome within any time frame.

• Involved a sample size of more than 50 that included CRC screening participation as an outcome.

• Involved only participants that are not up to date with screening.

### Search Strategy and Data Extraction

The systematic review was reported according to the Preferred Reporting Items of Systematic Reviews and Meta-Analyses (PRISMA) guidelines (20). Searches of five databases (PubMed, Embase, CENTRAL, PsycINFO, and CINAHL) were conducted for articles published from date of inception to June 2021. Literature search was performed using the search strategy in Supplementary Material.

A two-stage screening was adopted, title and abstract screening followed by full-text screening. At both stages, each reference was screened independently by two researchers with all discrepancies resolved by seeking the independent opinion of a third researcher.

Two researchers performed the data extraction with all disagreements resolved by mutual consensus.

### Quality Assessment of Included Articles

Quality control was performed by two researchers using the Cochrane Risk of Bias tool (21) which assesses five domains: bias arising from (1) the randomization process, (2) deviations from intended interventions, (3) missing outcome data, (4) measurement of the outcome and (5) bias in selection of the reported result. Data related to risk of bias was obtained during data extraction.

### Meta-Analysis

All analyses were conducted using R (version 4.1.0) using the meta package. For the studies included in the meta-analysis, the DerSimonian and Laird random effects model (22) was used to estimate the pooled risk ratios (RR) and their corresponding 95% confidence intervals (95% CI) for CRC participation. A RR of more than 1 indicates that the MI or tailored counseling participants had a higher risk of participating in CRC screening within the time frame as defined by the study compared to those in the comparator arm. A two-sided *P*-value of 0.05 was considered statistically significant. We assessed the statistical heterogeneity of the included studies’ results by chi-square test and I-squared statistic. We considered statistical heterogeneity to be significant when *p*-value of the chi-square test was 0.10 or if the I^2^ statistic was 50%. If there are sufficient studies, subgroup analysis was conducted separately on the primary outcomes for (1) duration of follow-up and (2) type of CRC screening modalities. This allows us to explore possible reasons for heterogeneity in pooled risk ratios.

To further minimize the heterogeneity between studies in the meta-analysis, whenever there were more than one non-MI comparator groups, the comparator group of each respective study was chosen if it contains the most similar non-MI components as compared to the MI group.

## Results

### Sample

**FIGURE 1:**
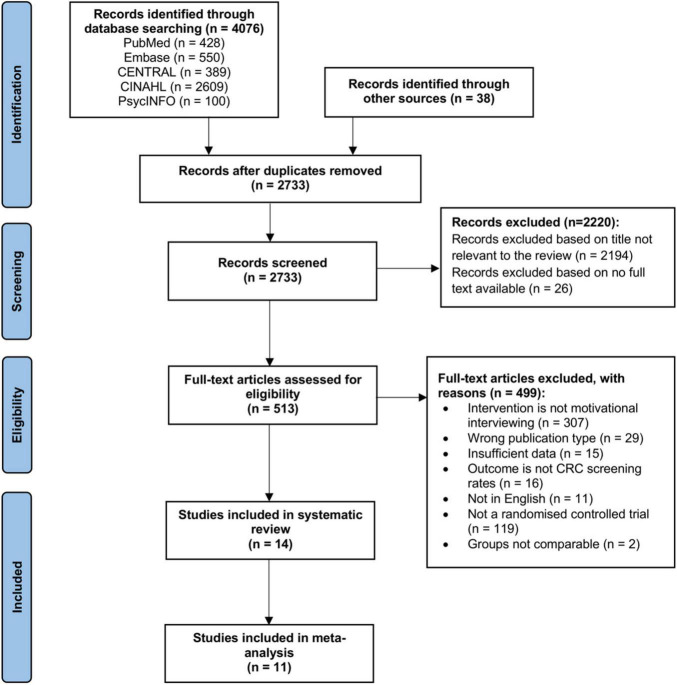
PRISMA flowchart of study selection.

Database searching and other sources retrieved 4,114 results. A total of 1,381 duplicates were removed. Title and abstract screening excluded a further 2,220 articles. Full text screening excluded 499 articles. 14 studies were eventually included in this systematic review. Out of the 14 studies, 11 studies were included for further meta-analysis. The PRISMA flowchart is presented in Figure 1. Detailed characteristics of the studies are reported in Table 1.

**TABLE 1:** Summary of study characteristics and findings.

Study	Study design	Participants	MI-containing intervention	Comparator group(s)	Outcome(s) of interest	Main findings
Adegboyega et al. (34)	RCT	50 and above, rural Appalachian resident with no history of CRC and no completion of CRC tests	**Intervention (MI)** 10-min MI session administered by the Lay Health Advisors (LHA) post-call action plan and a follow-up telephone MI session a week later	**Control** cancer screening brochures	Completion rate of CRC screening at either 3 or 6 months	No difference in the rate of CRC screening by study group (χ^2^=0.13,*p* = 0.72). 12% in the intervention group received CRC screening (*n* = 4 at 3 months), whereas 15% of those in the control group received CRC screening (*n* = 4 at 3 months, *n* = 2 at 6 months).
Arnold et al. (32)	RCT	Patients aged 50–75, without previous history of cancer, not up to date with CRC screening, does not have a first-degree relative with history of CRC	**Personal Call (PC) arm** FIT test and a brief literacy-informed educational intervention by the RA If no FIT kits returned in 4 or 8 weeks, health literacy and MI techniques will be used in a telephone call	**Automated Call (AC) arm** a FIT test and a brief literacy-informed educational intervention by the RA If no FIT kits returned, an automated telephone call with motivational messages received at 4 weeks and if needed again at 8 weeks after enrolment	FIT return rate within 12 months	No difference was found in the effectiveness of PC over AC. FIT return rate 69.2% in the AC arm, 67.0% in the PC arm. 9.4% in the PC arm returned FIT after the MI telephone call and 8.5% in the AC arm returned FIT after the automated telephone call.
Broc et al. (30)	RCT	50–74-year-old men and women with an average risk of CRC	**Telephone MI** a telephone interview with MI or a mailed FOBT test if uncontactable after 4 months No information or advice about CRC screening was given	**Computer-assisted individualized counseling (IC)** a computer-assisted telephone interview with the aid of a predefined algorithm prompter or a mailed FOBT test if uncontactable after 4 months Control (used in meta-analysis) reminder mail + FOBT	FOBT performed within 90 days	PP analysis: a 9.2% screening participation rate for controls (1,781/19,400), 18.8 and 19.9% for those that received some degrees of MI and IC, respectively (*p* = 0.001;*r* = 0.131;*OR* = 2.374), 29.5 and 31.3% for those that completed MI and IC interventions, respectively (*p* = 0.001;*r* = 0.219;*OR* = 4.321). ITT analysis: 10% screening rates for intervention groups combined vs. 9.2% for controls, 0.8% difference was significant (*p* = 0.001;*r* = 0.014;*OR* = 1.103). No difference was found between MI and IC on CRC screening participation rates.
Costanza et al. (27)	RCT	Patients between 50 and 75 years old with no colonoscopy in the last 10 years	**Telephone Counseling Call (TCC)** two-stepped intervention: a generic mailed print brochure, computer-assisted tailored telephone counseling call 3 months later MI included if subjects did not consider screening	**Control Group** Usual Care	Completion rate of any CRC screening test	No difference in screening rates between intervention and control arms.
Denis et al. (31)	RCT	Residents aged 50–74 who had not complied after two mailed invitations to visit their GP for CRC screening	**Telephone-based MI Intervention** Direct mailing of gFOBT kit based on responses during a MI telephone counseling call	**Computer-assisted telephone interview (CATI)** Direct mailing of the gFOBT kit based on responses during a computer-assisted telephone counseling call Control group* Direct mailing of the gFOBT kit + untailored recall letter reminding participants to visit their GP for CRC screening	gFOBT screening 1 year after intervention	ITT analysis: no difference was found in screening rates between intervention groups taken together (13.9%, 95% CI 13.5–14.4) and the control group (13.9%, 95% CI 13.4–14.4) at 1 year. PP analysis: no significant difference between CATI (517/2103, 24.6%, 95% CI 22.7–26.4) and MI groups (517/2192, 23.6%, 95% CI 21.8–25.4) (*P* = 0.44). Participants in the two intervention groups were significantly more likely to undergo screening (1034/4295, 24.1%, 95% CI 22.8–25.4) than in the control group (5359/41521, 12.9%, 95% CI 12.6–13.2) (*P* < 0.01).
Fortuna et al. (29)	RCT	Patients aged 50–74 overdue for CRC screening	**Letter + personal call** Letter + a personal telephone call using MI with assistance to schedule an appointment, provide referrals or mail a FIT kit	Reminder letter only* Letter that provided the contact detail of the outreach worker available to help schedule free screenings **Letter + autodial** Letter + a series of up to five automated telephone calls delivered on weeks 2, 8, 14, 28, and 38 for patients who are yet to screen **Letter + autodial + prompt** Letter + autodial + paper prompts delivered to treating clinician to remind the patient about overdue screening and facilitate discussion at a patient-initiated visit	CRC screening (FOBT, FIT, colonoscopy, flexible sigmoidoscopy, double contrast barium enema reports) 3 months after intervention	Letter + Personal Call group has a higher screening rate compared to letter alone for CRC (21.5% vs. 12.2%; AOR 2.0, 95% CI 1.1–3.9) Letter + Autodial + Prompt also has a higher screening rate than letter alone for CRC (19.6% vs. 12.2%; AOR 1.9, 95% CI 1.0–3.7) Letter + Autodial not effective at improving screening rates compared to a letter alone
Kinney et al. (24)	Cluster RCT	30–74-year old first degree relatives of CRC patients due for a colonoscopy	**TeleCARE** Educational brochure + tailored mail + telephone counseling with MI. Additional tailored letters with telephone call summary and action plan 1 week post-call	**Educational Brochure** Tailored to target population about family history and CRC risk, colonoscopy as recommended test	Medically verified colonoscopy at 9 months	PP: 35.4% (*p* = 0.001) in TeleCARE group had a colonoscopy by 9 months compared to 15.7% (*p* = 0.001) in the educational brochure group. ITT: TeleCARE group was almost 3 times more likely than control group to get screened (OR 2.83, 95% CI 1.87–4.28) (*p* = 0.001).
Lowery et al. (23)	RCT	Aged 21 and above first degree relatives of patients with CRC diagnosed under 60 years old due for a screening during the 2-year study period	**Tailored telephone counseling** Computer-assisted MI telephone interview based on participants’ responses to the baseline survey Post-call: mailed summary + a reminder postcard a month before their colonoscopy due date	**Mailed intervention** mailed letter + brochure	Colonoscopy screening reported in at least one of the follow-up mailed surveys at 6, 12 and 24 months with medically verified endoscopy reports	PP analysis: The prevalence of adherence for tailored and mailed intervention were 43.2 and 52.1% at baseline and were 54.0 and 49.8% at 24 months (*p* = 0.004). ITT analysis: 24% (unadjusted; HR, 1.24; *p* = 0.04) or 32% (adjusted; HR, 1.32; *p* = 0.01) increase in colonoscopy adherence at 24 months amongst those receiving the tailored telephone intervention.
Manne et al. (26)	RCT	Siblings of patients diagnosed with CRC less than 61 years old	**Tailored Print + Telephone Counseling (TP + TC)** Mailed tailored print + a telephone counseling session a week later using MI	Tailored Print (TP)* Mailed personalized cover letter and booklet tailored specifically to participants’ survey + a tailored print newsletter a month after **Generic print (GP)** Mailed non-tailored cover letter and educational pamphlet, not specific for at-risk populations	Colonoscopy or FS and FOBT 6–8 months after baseline	No significant increase in screening rate in TP+TC group compared to TP group All 3 groups of immediate siblings had increased screening adherence. The TP (ITT results: 24.8%) and TP + TC group (ITT results: 25.9%) had significantly higher CRC screening adherence than the GP group (ITT results: 13.7%). Those in the combined tailored intervention (Wald Chi-square 6.97, *p* = 0.008) were 2.12 times more likely to adhere to screening than those in the GP group.
Menon et al. (28)	RCT	Participants 50 years or older; having no personal or family history of CRC, non-adherent to screening	**MI** a single, telephone-based MI Follow-up interviews at 1 and 6 months post intervention	**Tailored Counseling** Trained interventionists read computer-generated tailored messages to participants via phone interview Control* Usual care	Completion of any screening test (stool blood test, sigmoidoscopy, or colonoscopy) within 12 months of the intervention	Interventions not significantly associated with greater probability of screening compared with usual care while tailored counseling was significantly more effective than control (*p* = 0.02), no significant difference than MI. Proportion who completed a CRC screening test post-intervention was 11.8% (usual care), 23.8% (tailored counseling), and 18.5% (motivational interview). Participants in the tailored counseling group had 2.2 times the odds of completing post-intervention CRC screening than did the participants in the usual-care group (AOR 2.2, 95% CI 1.2–4.0).
Salimzadeh et al. (25)	RCT	First-degree relatives (FDRs) of patients diagnosed with CRC under the age of 60, due for a colonoscopy	**MI counseling** One-time, phone-based MI counseling by a trained oncology nurse	**Control group** One 15-20-min non-tailored telephone interview by a physician providing general CRC screening information	Completion of colonoscopy within 6 months	83.5% screening attendance in the intervention vs. 48.2% in the control group (COR = 5.4; 95% CI 2.9–10.0) Significantly higher proportion of participants with correct answers to CRC knowledge statements as compared to the control group
Vernon et al. (33)	Stepped randomized trial	Vietnam-era U.S. military Veterans 50 years old and above overdue for CRC screening	*Step 2 for participants with no CRC screening after Step 1—automated telephone or telephone call or mailed letter, followed up 9 months later* **Step 2 Automated MI telephone call** Information on CRC screening provided, with tailored messages based on responses Step 2 Counselor-delivered brief MI telephone call* Provide information about CRC rates and risk, benefits of being screened, tests - colonoscopy and FOBT	Step-2 control group* Only a follow-up survey after 9 months to collect data on CRC screening completion (same for all Step 2 groups)	Self-reported completion of screening 9 months after each step and 18 months after both steps	No difference between Step 1 intervention groups and control. ITT analysis: no significant difference in CRC screening completion at Step 2 between either of the Step 2 intervention groups and Step 2 control group. Automated and counselor-delivered MI showed 27.9 and 30.7%, respectively, compared to 23.1% in Step 2 control group. PP analysis: statistically significant difference in CRC screening completion between either of the Step 2 intervention groups and Step 2 control group. Automated and counselor-delivered MI showed 34.5 and 34.7%, respectively compared to 22.6% in Step 2 control group. Significant increase in CRC screening in both ITT and PP analysis for participants who received any combination of Step 1 and Step 2 interventions compared to survey-only controls, no individual combination showed significant increase in CRC screening.
Turner et al. (35)	RCT	Patients who missed more than 75% of their primary care appointments, with a colonoscopy scheduled at designated endoscopy suites, not ready to attend the appointment	**Peer coaches with MI** MI call from peer coaches within 2 weeks of participants’ scheduled colonoscopy appointment	**Control** 2 brochures	Rate of colonoscopy appointment attendance within 2 weeks	Peer coach intervention group had over two-fold greater adjusted odds ratios (2.14, 95% CI 0.99–4.63, *p* = 0.05) of attending colonoscopy compared to the brochure group and there’s an absolute difference of 11% for colonoscopy attendance (Peer coach: 68.6%, Brochure: 57.6%, *p* = 0.18) between the two groups.
Lasser et al. (36)	RCT	Patients aged 52–74 years old not up-to-date with CRC screening	**Patient navigation intervention** a reminder letter, brochure, maximum 6 h of navigation with MI techniques across 6 months Help was offered to schedule colonoscopies, refer patients to insurance, emotional support offered by accompanying patients to the appointment	**Control** Usual care	Completion of any CRC screening tests within 1 year based on medical records	33.6% of the intervention group vs. 20.0% of the control group underwent screening by 1 year (*p* = 0.001). For those randomized to receive intervention, screening rate was 39.8% for those able to be contacted and only 18.6% for those uncontactable (*p* = 0.001).

CRC, colorectal cancer; MI, motivational interviewing; RCT, randomized controlled trial; FDR, first-degree relative; PC, personal call; AC, automated call; IC, individualized counseling; CATI, computer-assisted telephone interview; TP, tailored print; TC, telephone counseling; FIT, fecal immunochemical test; (g)FOBT, (guaiac) fecal occult blood test; PP, per-protocol; ITT, intention-to-treat; (A/C)OR, (adjusted/crude) odds ratio; CI, confidence interval; HR, hazard ratio; GP, general practitioner; US, United States. *Comparator group used in the meta-analysis for studies with more than one comparator group.

### Characteristics of Included Studies

#### Participants and Setting

The inclusion criteria for participants differed across studies. Three of the studies (23–25) only included those with at least one first-degree relative (FDR; sibling, parent or child) diagnosed with CRC and one study included only siblings of CRC patients (26). Five studies specified FDR as an exclusion criterion (27–32). With the exception of Lowery et al. (23), all studies (24–36) excluded participants who were up to date with CRC screening. The age of the study participants ranged from 30 to 75 years old.

The included studies were published between 2007 and 2020 and were conducted in the United States (*n* = 11), France (*n* = 2) and Iran (*n* = 1).

#### Motivational Interviewing Intervention Delivery

MI was delivered via telephone in all included studies except for Adegboyega et al.’s study which delivered the MI sessions face-to-face (34). The professions of MI interventionists varied across the studies, including nurse or psychologists (25, 30, 31), unspecified healthcare workers (34, 36), peer coaches (35), genetic counselors (24), outreach workers (29), health educators (26), prevention counselors (32), and counselors of unspecified professions (23, 27, 28, 33). Frequency of MI sessions ranged from one to two times over the course of the study, with the exception of Lasser et al.’s study, totaling up to a maximum of 6 h within 6 months (36).

Components of MI that were incorporated varied across the studies. Mentions of various MI components were included such as the evocation of change talk (25, 28, 30, 31, 33, 36), the elicit-provide-elicit framework when providing information (24, 25, 28, 30, 31, 33, 35) and exploring ambivalence (25, 28, 30, 32, 34).

In terms of the structure of MI counseling sessions, four studies adhered to a scripted guide for interventionists (26, 27, 35, 36), two provided prompts to follow (24, 28), one used computer-assisted software (23) and two used a free interview format (30, 31). After the MI session ended, five studies provided a post-call summary and/or action plan and/or reminder (23, 24, 26, 33, 34). One study made no explicit elaboration of how MI was structured (29).

Additionally, ten studies (23, 24, 26, 27, 29, 31–34, 36) used MI in conjunction with one or more active interventions. Out of those, six studies included print material mostly before MI (24, 26, 27, 29, 33, 36) and two studies mailed Fecal Immunochemical Test (FIT) or FOBT test kits before (32) or after MI (31).

#### Motivational Interviewing Intervention Training and Intervention Fidelity

MI interventionists received different levels of training across the RCTs which will impact the validity of study findings. Three studies (29, 30, 32) reported little to no details of MI intervention training. Trainings typically included didactic sessions, role-playing (27, 28, 31, 34–36) and focused on equipping interventionists with the skills to use open-ended questions, express empathy, practice reflective listening, explore, and resolve ambivalence, assess readiness to change, promote self-efficacy and communicate cancer risks (24, 25, 28, 33–36). Training duration was reported to range from half a day to four full days (25, 27, 28, 31, 33–36).

In addition to training, assessing intervention fidelity is crucial to affirm the quality and consistency of MI delivery. Nine studies reported fidelity assessments within the retained articles or elsewhere (23, 24, 26–28, 31, 33, 34, 36). Namely, the assessments were conducted by audiotaping live or roleplaying sessions which are subsequently coded and evaluated by MI experts or supervisors. Feedback was provided based on predetermined criteria or checklist (26, 27). Three studies (24, 28, 34) reported elsewhere the Motivational Interviewing Treatment Integrity (MITI) as a coding system (37) to measure MI fidelity.

#### Non-motivational Interviewing Comparator Groups

The non-MI comparator groups of the retained studies mainly involved non-tailored or tailored call (28, 29, 32, 33), non-tailored or tailored print material such as brochure or letter (23, 24, 26, 29–31, 33–35), a combination of the above (29, 33) or usual care (27, 28, 33, 36). Eight studies had one non-MI comparator group (23–25, 27, 32, 34–36) while the remaining six studies had more than one non-MI comparator group (26, 28–31, 33).

### Risk of Bias Assessment

**FIGURE 2:**
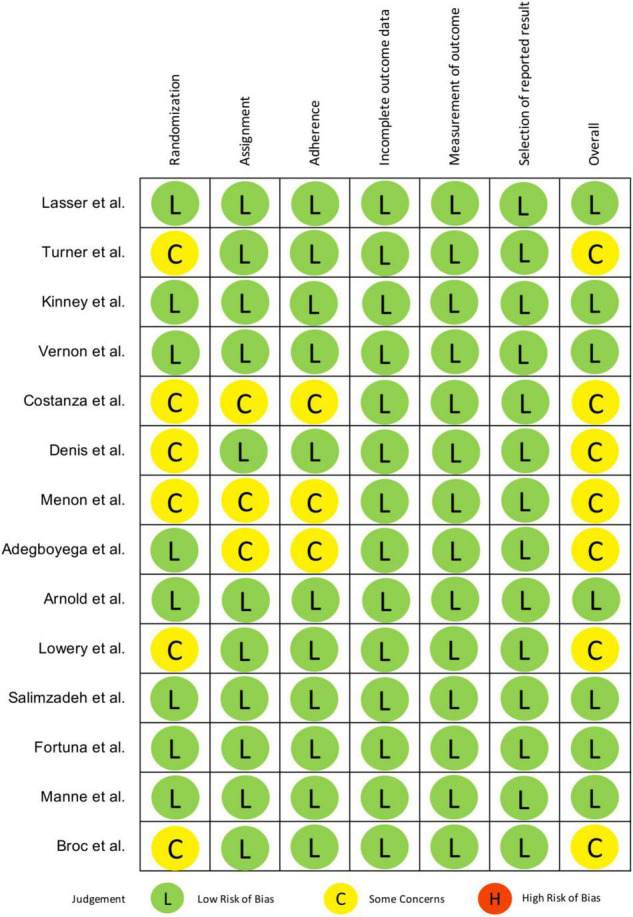
Risk of bias assessment.

Figure 2 reports methodological quality of retained studies in this systematic review using the Cochrane method to assess risk of bias in RCTs. The biases assessed include selection bias due to inadequate generation of a randomized sequence and/or allocation concealment before assignment, performance bias due to knowledge of allocation by participants and study personnel, detection bias due to knowledge of allocation by outcome assessors, attrition bias due to incomplete outcome data and reporting bias due to selective reporting of outcome. A judgment of the risk-of-bias level, namely (1) low, (2) some concerns or (3) high, was reached for each domain based on the Cochrane criteria and algorithms (21).

There were some concerns of selection bias due to the lack of reporting of allocation concealment (27, 30, 31) and differences in baseline characteristics (23, 28, 35). Ten studies reported intention-to-treat (ITT) analysis (23–26, 29–31, 33, 35, 36) while three studies only performed per-protocol (PP) analysis (27, 28, 34). Arnold et al. (32) didn’t explicitly report ITT analysis but was implied, thus it was judged to have low risk of performance bias. Blinding of outcome assessors to intervention assignment was reported in three studies (24, 29, 36). Four studies used self-reported data to measure screening rates (26, 33, 34), cross-checking of medical records was only performed in two of them (23, 26). Despite this, knowledge of intervention received was deemed unlikely to have influenced reporting of one’s screening status. Therefore, detection bias in terms of outcome measurement was rated as low in all.

### Overall Effect of Motivational Interviewing Effects on Colorectal Cancer Screening Uptake

Eight studies (23–26, 29, 30, 35, 36) lent support to MI-containing interventions increasing CRC screening uptake as compared to another comparator group. Two of those studies, however, demonstrated no statistically significant difference in outcome between MI and tailored print material (26) or computer-assisted telephone counseling (30). The remaining six studies found no statistically significant differences in screening rates in the MI intervention group compared to control and other non-MI comparator groups (27, 28, 31–34).

### Meta-Analysis of the Effect of Motivational Interviewing Intervention on Colorectal Cancer Screening Uptake

We performed separate meta-analysis of risk ratios amongst ITT and PP data from studies. Studies which reported both ITT and PP data were included in both meta-analyses. For studies that have more than one non-MI comparator arm, the comparator arm containing the most similar non-MI components as the MI-containing intervention arm was used in this meta-analysis where possible. Since Vernon et al.’s study (33) has more than one MI intervention arm, the stepped intervention involving Step 2 counselor-delivered MI after Step 1 mailed letter was used. Mailed letter, as a non-MI component, was commonly found to be a part of the intervention and control groups of other studies in this meta-analysis, therefore, it was chosen over other Step 1 interventions detailed in Table 1. To balance the non-MI components of the chosen MI stepped intervention in Vernon et al.’s study (33), Step 2 survey-only control group who has received mailed letter in Step 1 was identified as the control arm in this meta-analysis. Only three studies (26, 29, 33) have a non-MI comparator arm with identical non-MI components as the MI intervention group which can be helpful for isolating the effects of MI.

Three studies in the systematic review were excluded from the meta-analysis as they did not fulfill the selection criteria for meta-analysis. Arnold et al. (32) included MI components in its non-MI comparator arm. Adegboyega et al. (34) had less than 50 participants that included CRC screening participation as an outcome due to high attrition rates. Lastly, Lowery et al. (23) used the percentage increase in screening adherence to measure outcome instead of the absolute screening rate adopted in all other studies.

#### Intention-to-Treat Analysis

**FIGURE 3:**
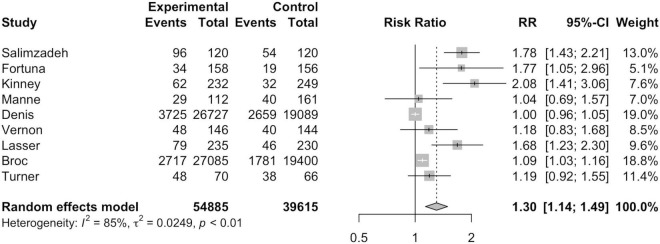
Intention-to-treat analysis forest plot.

As seen in Figure 2, nine studies were included in ITT analysis including a total of 54,885 MI participants and 39,615 non-MI controls. Broc et al. (30) and Denis et al. (31) combined MI and another telephone intervention group together in their ITT analyses, thus, the combined data for the respective intervention groups is reflected in Figure 3. Overall, ITT analysis found that MI participants exhibited a significantly higher risk of CRC screening participation than non-MI controls (*RR* = 1.30; 95%*CI* = 1.14, 1.49; *I*^2^=85.0%; *p* = 0.01).

#### Per-Protocol Analysis

**FIGURE 4:**
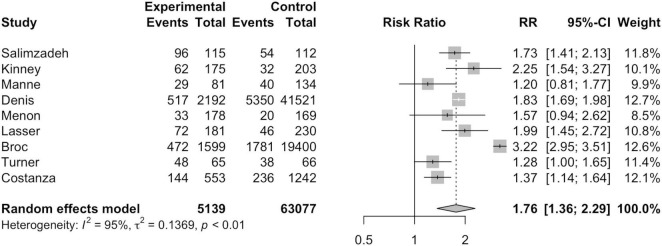
Per-protocol analysis forest plot.

As seen in Figure 4, nine studies were included in per-protocol analysis including 5,139 MI participants and 63,077 non-MI controls. Broc et al. (30) and Denis et al. (31) analyzed the data of those who refused intervention or failed to be contacted due to technical reasons together with the control group in PP analysis. MI participants exhibited a significantly higher risk of CRC screening participation than non-MI controls, notably greater than that in ITT analysis (*RR* = 1.76; 95%*CI* = 1.36, 2.29; *I*^2^=95.0%; *p* = 0.01).

#### Subgroup Analysis: Outcome Measure Time Frame

Subgroup analysis on outcome measure time frame was also conducted on nine studies with ITT analysis and nine studies with PP analysis, as shown in Table 2. Costanza et al. (27) did not provide information on the exact timeline of data collection and is thus excluded from PP time frame subgroup analysis. Overall, there is no significant difference between outcome measure time frame and CRC screening uptake in either ITT (*p* = 0.3022) or PP analysis (*p* = 0.8390).

**TABLE 2:** Subgroup differences in screening rates stratified by outcome measure time frame.

Screening rate measured *x* months after intervention	Studies	Subgroup total (*N*)	Risk (95% CI)	*I*^2^ (%)	Test for subgroup effect (*p*-value)
**Intention-to-treat (ITT) analysis**					
≤3 months	Fortuna et al. (29), Broc et al. (30), and Turner et al. (35)	3	1.1737 (0.9835, 1.4008)	45.1	0.3022
3 < *x* < 12 months	Kinney et al. (24), Salimzadeh et al. (25), and Manne et al. (26)	3	1.5979 (1.1266, 2.2662)	69.5	0.3022
≥12 months	Denis et al. (31), Vernon et al. (33), and Lasser et al. (36)	3	1.2268 (0.8855, 1.6997)	81.8	0.3022
**Per-protocol (PP) analysis**					
≤3 months	Broc et al. (30) and Turner et al. (35)	2	2.0470 (0.8318, 5.0374)	97.8	0.8390
3 < *x* < 12 months	Kinney et al. (24), Salimzadeh et al. (25), and Manne et al. (26)	3	1.6851 (1.2511, 2.2696)	61.7	0.8390
≥12 months	Menon et al. (28), Denis et al. (31), and Lasser et al. (36)	3	1.9331 (1.6988, 1.9781)	0.0	0.8390

*CI = confidence interval.*

#### Subgroup Analysis: Colorectal Cancer Screening Modalities

As shown in Table 3, subgroup analysis on CRC screening modalities was conducted on 11 studies in ITT analysis and nine studies in PP analysis. In ITT analysis, there was a statistically significant difference between CRC screening participation and acceptable screening modalities. There is also a statistically significantly higher risk of CRC screening participation when a mixture of different screening modalities was offered and accepted as a screening outcome (*RR* = 1.4914; 95%*CI* = 1.1612, 1.9156; *I*^2^=24.1%; *p* = 0.0044). Studies that accepted colonoscopy (*RR* = 1.4736; 95%*CI* = 1.0985, 1.9769; *I*^2^=72.9%; *p* = 0.0044) were superior to FIT/FOBT only (*RR* = 1.0439; 95%*CI* = 0.9576, 1.1380; *I*^2^=82.0%; *p* = 0.0044). In PP analysis, there was no significant difference between screening participation and any CRC screening modalities.

**TABLE 3:** Subgroup differences in screening rates stratified by CRC screening modalities.

CRC screening modality	Studies	Subgroup total (*N*)	Risk (95% CI)	*I*^2^ (%)	Test for subgroup effect (*p*-value)
**Intention-to-treat (ITT) analysis**					
Colonoscopy	Kinney et al. (24), Salimzadeh et al. (25), Manne et al. (26), and Turner et al. (35)	4	1.4736 (1.0985, 1.9769)	72.9	0.0044
FIT/FOBT only	Broc et al. (30) and Denis et al. (31)	2	1.0439 (0.9576, 1.1380)	82.0	0.0044
Mixed	Fortuna et al. (29), Vernon et al. (33), and Lasser et al. (36)	3	1.4914 (1.1612, 1.9156)	24.1	0.0044
**Per-protocol (PP) analysis**					
Colonoscopy	Kinney et al. (24), Salimzadeh et al. (25), Manne et al. (26), and Turner et al. (35)	4	1.5608 (1.2161, 2.0033)	65.3	0.3421
FIT FOBT only	Broc et al. (30) and Denis et al. (31)	2	2.4253 (1.3963, 4.2124)	98.9	0.3421
Mixed	Costanza et al. (27), Menon et al. (28), and Lasser et al. (36)	3	1.5863 (1.2291, 2.0474)	50.8	0.3421

*CRC, colorectal cancer; CI, confidence interval; FIT, fecal immunochemical test; FOBT, fecal occult blood test.*

## Discussion

### Significance of Review

This review substantiates findings from two similar reviews on MI effects in health screenings (38, 39). Mixed evidence is shown for the effectiveness of MI on improving colorectal screening uptake. Four studies (26–29) on CRC screening uptake in the review by Miller et al. (38) and three studies in the review by Chan and So (39) were also included in this study. To the best of our knowledge, no systematic review has evaluated the effectiveness of MI strictly on improving CRC screening only.

### Discussion of Findings

#### Meta-Analysis Findings

Out of the 11 studies included for this meta-analysis, ITT and PP analysis were conducted for nine studies each. Both ITT and PP analysis found a statistically significantly higher screening rate in participants of MI intervention groups. Compared to ITT analysis, higher risk of CRC screening participation was found in MI participants than non-MI controls under PP analysis. This can be attributed to the exclusion of those who were not followed up or could not be reached by telephone since MI delivery can often result in low rates of technical success (26, 31). For instance, Denis et al. (31) found that only 33.6% of the calls were successful.

Subgroup analysis showed no statistically significant difference between timing of measuring CRC screening rates and CRC screening participation. However, the difficulty in finding an optimal time frame for data collection should still be acknowledged. For instance, having a longer follow-up time frame may allow more time for people to schedule and complete screenings that require a clinic visit such as colonoscopy. Meanwhile, data collection and intervention cannot be too far apart as MI effects may have weaned off after a certain amount of time. Attrition rates could also be higher in studies with a longer follow-up period. On the other hand, having a shorter follow-up period may not reflect MI effects as many participants may not have undergone the intervention yet. More research is needed to ascertain the effects of data collection timeframe on MI intervention effects to evaluate existing and future literature of CRC screening.

Additionally, a subgroup analysis was conducted on the different CRC screening modalities used. In PP analysis, none of the CRC screening modalities alone or in combination were significantly better at increasing CRC screening uptake. It is possible that those who have undergone MI interventions were already convinced of the benefits of CRC screening and understood the different screening modalities regardless of the screening tests offered. In ITT analysis, studies that accepted a mixture of screening tests post-MI intervention were significantly better at increasing CRC screening uptake than those that only accepted colonoscopy or FIT/FOBT. This finding is in contrast to Chan and So’s theories (39) postulating that people may have more questions when presented with various screening options and thus may not resolve their ambivalence and decide to screen. For all three studies in ITT analysis that accepted a mixture of screening tests, MI interventions mainly focused on helping participants to decide between colonoscopy and FIT/FOBT (29, 33, 36). It is possible that the door-in-the-face psychological phenomenon (40) was at play whereby the participants rejected the initial request of going for colonoscopy but felt guilty to turn down the MI interventionist’s alternative offer of a less invasive screening test which is FIT/FOBT. The participants who were reluctant to go for a more invasive test like colonoscopy may be more likely to comply with FIT/FOBT as compared to being offered colonoscopy or FIT/FOBT alone. Additionally, MI-containing interventions that offered FIT/FOBT alone had a significantly lower impact on CRC screening uptake than those that offered mixed screening modalities. It is possible that the average person may have already been willing to comply with the non-invasive tests without requiring MI interventions.

#### Qualitative Findings

Overall, there is mixed evidence to support whether MI promotes compliance of CRC screening. The different benefits of MI were cited in various studies included in this review (23, 25, 29, 31, 35). Typically for those reluctant to screen, MI can help clients resolve ambivalence themselves and elicit their own intrinsic motivation which can be more effective than traditional methods (41).

However, the segment of MI that explores ambivalence might have enhanced participants’ lack of motivation for screening rather than engendering changes in screening behavior. Two studies (28, 30), in particular, have highlighted the greater emphasis placed on the “exploring ambivalence” segment of MI. According to Miller and Rollnick (42), MI should be “eliciting the client’s own change talk and taking care not to reinforce counter-change talk.” Resolving ambivalence—differentially evoking change talk (pro-change arguments) while respectfully responding to sustain talk (anti-change arguments)—is also necessary in order to evoke one’s intrinsic motivation to change (13, 14, 41). MI is not to be confused with decisional balance, where equal attention is devoted to both reasons to screen and not to screen (43). This clarification of the original conception of MI was published in 2009, thus, some of the earlier studies in this review (23, 26–28, 35) may have incorporated the MI techniques differently.

### Limitations of This Review and Meta-Analysis

This study is not without its limitations. It cannot be ruled out that there may be publication bias involved as negative data are generally less likely to be published. Researchers in this study also did not conduct additional searches after June 2021. Additionally, the literature search was limited to only five databases and publications written in languages other than English were excluded.

Moreover, there is a great level of heterogeneity among included studies. Only nine studies (23, 24, 26–28, 31, 33, 34, 36) assessed or reported treatment fidelity. Two studies (28, 31) have also cited that some MI counselors were more proficient in delivering MI than others. Although there has been some development in fidelity scales and measurements (44), more research is needed to standardize fidelity assessment of MI delivery to ensure MI efficacy can be compared effectively across studies. Given that there were variations in MI protocols across the studies, it is possible that they may also differ across different countries (45). Therefore, more research is needed to explore cross-cultural adaptations of MI protocol and fidelity scale to improve our understanding of the quality and consistency of MI delivered to the participants. Fidelity measurement tools should also include assessments of MI-specific characteristics to allow definitive differentiation from other therapeutic approaches such as supportive counseling (46). Additionally, the baseline characteristics of participants in some studies make it difficult to draw objective conclusions. For instance, two studies (23, 30) admitted that participants who took part were already more motivated to participate in screening or had more women, non-disadvantaged individuals in the MI group. Such individuals have been found to be significantly more likely to go for CRC screening (47–49), potentially displaying selection bias unreflective of people in the general population. Together, these may be factors that play a part in increasing heterogeneity and inter-study variance (50, 51).

The studies included in the meta-analysis have an *I*^2^ of 85 and 95% under ITT and PP analysis, respectively. Random effects model was utilized as opposed to a fixed effects model to address the substantial heterogeneity among included studies. Studies in the meta-analysis were also divided into subgroups to investigate reasons behind the heterogeneity. As much as possible, wherever MI was bundled into other interventions, a non-MI comparator group with the most similar non-MI components as the MI group was used in the meta-analysis. This allows us to examine and compare MI effects in isolation across the various studies. However, in the end, only three studies (26, 29, 33) had identical non-MI components in its MI-containing arm and non-MI comparator arm. Therefore, findings in this review and meta-analysis should be interpreted with caution and not be considered as definitive assertions.

### Implications for Future Practice

Although the meta-analysis showed that MI-containing interventions were effective in increasing CRC screening uptake, more research is needed to compare the effects of different combinations of MI and other CRC screening promotion strategies. MI may be an effective CRC screening strategy especially when delivered in full, compared to other modalities, however, higher cost may be involved due to the multiple telephone attempts and training required for interventionists (52, 53). Thus, the trade-off between costs, effort and the marginal benefits of MI must be carefully considered, especially in communities with limited and sparse resources.

More efforts would also be helpful to ascertain whether offering a range of CRC screening modalities during MI is beneficial for CRC screening compliance. Future research may consider tailoring advice of screening modalities based on CRC risk levels as it can also be an important consideration for people’s screening decisions on top of the invasiveness and logistical convenience of the tests.

## Conclusion

Overall, this systematic review and meta-analysis showed mixed evidence for the effectiveness of MI on CRC screening. Its efficacy can be highly variable depending on participant characteristics and interventional delivery. Meta-analysis found no significant effect of follow-up duration on MI efficacy, yet a statistically significant effect was found when a mixture of screening modalities was offered during MI and accepted as a screening outcome. However, one is advised to exercise caution when interpreting the findings due to the small sample size and heterogeneity of the included studies. Future research should also consider better ways to quantify the efficacy of MI as a stand-alone versus a supplementary screening promotion strategy to increase CRC screening adherence.

## Data Availability Statement

The original contributions presented in the study are included in the article/Supplementary Material, further inquiries can be directed to the corresponding author/s.

## Author Contributions

CH and AL contributed to conception and design of the study. NL, ML, and AL conducted the literature search. NY and NK performed the data extraction. AL performed the statistical analysis. NL and ML wrote the first draft of the manuscript. NL, ML, AL, NY, NK, and CH wrote sections of the manuscript. All authors contributed to manuscript revision, read, and approved the submitted version.

## Conflict of Interest

The authors declare that the research was conducted in the absence of any commercial or financial relationships that could be construed as a potential conflict of interest.

## Publisher’s Note

All claims expressed in this article are solely those of the authors and do not necessarily represent those of their affiliated organizations, or those of the publisher, the editors and the reviewers. Any product that may be evaluated in this article, or claim that may be made by its manufacturer, is not guaranteed or endorsed by the publisher.
