# Supplementary material for: Motivational Interviewing to Improve the Uptake of Colorectal Cancer Screening: A Systematic Review and Meta-Analysis
Source: Front Med (Lausanne). 2022 Apr 26;9:889124. doi: 10.3389/fmed.2022.889124 (PMC9090440; doi:10.3389/fmed.2022.889124)
Supplement: Supplementary file 1 [file Table_1.DOCX]

Supplementary Material

# Search Strategy

The search terms were constructed based on domains of population, intervention, comparator, outcome and study designs (PICOS) as **Supplementary** **Table 1**. Subsequently, these search terms were combined using Boolean operator OR within the same domains, and “AND” Boolean operator between domains of PICO as described in **Supplementary Table 2**.

**Supplementary Table 1. Population, interventions, comparators, outcomes and study designs (PICOS) of this study**

| **PICOS** | **Criteria** |
| --- | --- |
| Problem/Patient/Population | colorectal cancer prevention |
| Intervention | Any intervention incorporating motivational interviewing as part of any arm, delivered in any format |
| Comparator | Any comparator that did not involve motivational interviewing such as tailored telephone counselling, usual care, tailored print, reminder letter |
| Outcome | colorectal cancer screening uptake |
| Study Designs | Randomised controlled trials |

**Supplementary Table 2. Search terms and number of results in each database**

| **Database** | **Search Term** | **Results (N)** |
| --- | --- | --- |
| PubMed | ((colorectal cancer OR (colorectal AND cancer) OR colon cancer OR (colon AND cancer) OR rectal cancer OR (rectal AND cancer)) AND (motivational interview* OR (tailor* AND counsel*) OR tele*) AND screening AND (random* or trial)) | 428 |
| EMBASE | (((colorectal AND cancer OR colon) AND cancer OR (colon AND cancer) OR rectal) AND cancer OR (rectal AND cancer)) AND (motivational AND interview* OR (tailor* AND counsel*) OR tele*) AND screening AND (random* OR trial) | 550 |
| CENTRAL | ((colorectal cancer OR (colorectal AND cancer) OR colon cancer OR (colon AND cancer) OR rectal cancer OR (rectal AND cancer))[All Text] AND (motivational interview* OR (tailor* AND counsel*) OR tele*) [All Text] AND screening [All Text] AND (random* OR trial) [All Text] | 389 |
| CINAHL | TX ((colorectal cancer OR (colorectal AND cancer) OR colon cancer OR (colon AND cancer) OR rectal cancer OR (rectal AND cancer)) AND (motivational interview* OR (tailor* AND counsel*) OR tele*) AND screening AND (random* OR trial) | 2609 |
| PsycINFO | ((colorectal cancer OR (colorectal AND cancer) OR colon cancer OR (colon AND cancer) OR rectal cancer OR (rectal AND cancer)) AND (motivational interview* OR (tailor* AND counsel*) OR tele*) AND screening AND (random* or trial)) | 100 |
